# A phase II study of ENMD-2076 in advanced soft tissue sarcoma (STS)

**DOI:** 10.1038/s41598-019-43222-6

**Published:** 2019-05-14

**Authors:** Zachary Veitch, Alona Zer, Herbert Loong, Samer Salah, Maryam Masood, Abha Gupta, Penelope A. Bradbury, David Hogg, Andrew Wong, Rita Kandel, George S. Charames, Albiruni R. Abdul Razak

**Affiliations:** 1 Princess Margaret Cancer Centre, Toronto, Canada; 2 Department of Medicine, University of Toronto, Toronto, Canada; 3 Department of Pathology and Lab Medicine, Mount Sinai Hospital, Toronto, Canada; 4 Department of Lab Medicine and Pathobiology, University of Toronto, Toronto, Canada; 5 Lunenfeld-Tanenbaum Research Institute, Sinai Health System, Toronto, Canada

**Keywords:** Cancer genomics, Sarcoma

## Abstract

ENMD-2076, an aurora-A kinase inhibitor with anti-angiogenic properties, has shown activity in solid and hematologic malignancies. We investigated oral ENMD-2076 in an open-label, single-arm phase II study using 275 mg daily on a 28-day cycle in patients with advanced soft-tissue sarcomas (STS) receiving ≤1 line of prior therapy. Primary endpoint was 6-month progression-free survival (PFS) with ≤15% indicating no interest, and ≥40% indicating further interest in ENMD-2076. Secondary/exploratory endpoints included clinical benefit (CBR ≥6-months) and objective response (ORR) rates, PFS, OS, safety, and whole-exome sequencing (WES) for potentially associated biomarkers. Overall, 23/25 (92%) patients receiving ENMD-2076 were efficacy evaluable with median follow-up of 14 months (range 2.2–39.5). Common subtypes were leiomyosarcoma (n = 10), undifferentiated pleomorphic sarcoma (n = 3), angiosarcoma (n = 3), and alveolar soft-part sarcoma (n = 3). The 6-month PFS was 20.8% (95% CI:3.2–38.4) with a CBR of 17% (95% CI:1.55–33.23) and ORR of 9% (95% CI:3.08–20.46). Median PFS was 2.5 months (95% CI:2.20–4.47) and OS was 14.1 months (95% CI:6.07–20.07). The most common high-grade treatment-related adverse event was hypertension (60%). WES identified *PTPRB* mutations in 3/4 patients (p = 0.018) benefiting from ENMD-2076. Although this study failed to meet its primary endpoint, occasional responses and prolonged stable disease was noted. ENMD-2076 evaluation in *PTPRB* mutated tumors and/or angiosarcoma is warranted.

## Introduction

Soft tissue sarcomas (STS) are a heterogenous group of rare mesenchymal tumors with a wide range of biological behavior, molecular phenotypes, prognosis, and response to systemic treatment^1^. Cytotoxic chemotherapy remains a mainstay for advanced STS providing an objective response rate (ORR) of approximately 25% and a median overall survival (OS) of 10–18 months^2^. As limited benefit is seen from current treatments, a clinical need exists for therapies with improved efficacy and safety profiles.

Several anti-angiogenic agents have been explored for STS in clinical trials. Although bevacizumab demonstrated limited benefit in metastatic STS^3^, the vascular endothelial growth factor (VEGFR 1/2/3) and platelet-derived growth factor (PDGFR A/B) receptor antagonist pazopanib has shown progression-free survival (PFS) improvement in a phase III trial compared to placebo for STS^4^. The anti-PDGFR-A antibody olaratumab in combination with doxorubicin also demonstrated improved OS compared to doxorubicin alone in a phase II trial for STS^5^. However, a confirmatory phase III trial failed to validate this benefit^6^ highlighting the difficulty of identifying efficacious therapies in this heterogenous group.

The aurora kinase family of serine/threonine kinases, specifically aurora kinase A (AURKA), play a key role in cellular division. AURKA is involved in centrosome maturation, bipolar spindle assembly, and chromosome separation^7^, with inhibition leading to mitotic delay and cell death^8^. ENMD-2076 (CASI Pharmaceuticals Inc.) is a novel, oral small molecule multi-kinase inhibitor of AURKA, as well as VEGFRs, fibroblast growth factor receptors (FGFRs), FMS-like tyrosine kinase (Flt3) and c-kit^9^. This novel compound has demonstrated single agent activity in both solid^10^ and hematologic^11^ malignancies in early phase clinical trials^12^; including one patient with alveolar soft-part sarcoma (ASPS) who gained clinical benefit with disease stabilization for 21 months^13^.

The purpose of this study was to assess the activity and safety of ENMD-2076 monotherapy in treatment-naïve, or early treatment (≤1 treatment line) metastatic STS patients. We also aimed to evaluate tumor genomic alterations as predictive biomarkers to ENMD-2076.

## Results

**Figure 1:**
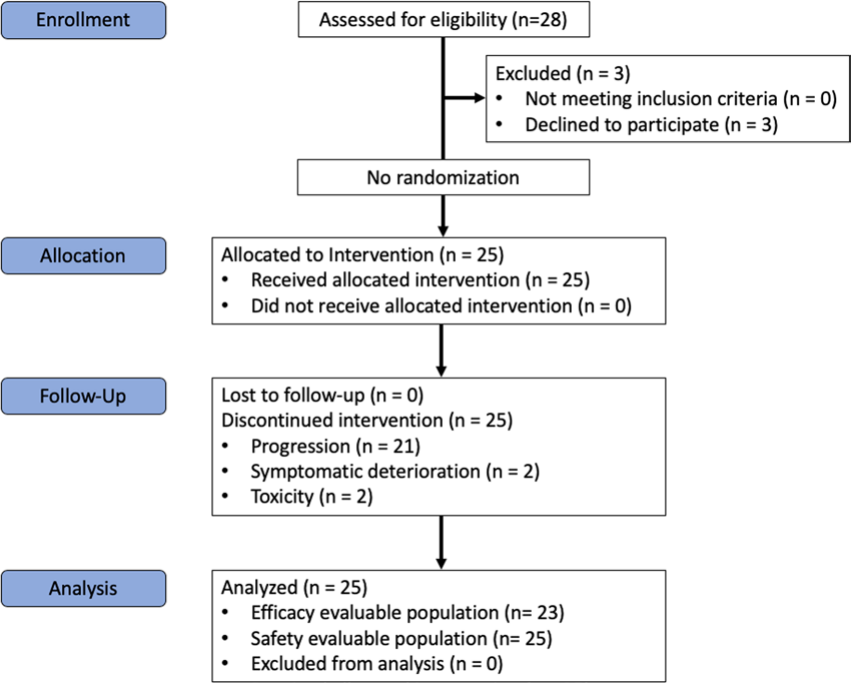
Consort diagram for ENMD-2076 phase II study.

At time of data cut-off (August 17^th^, 2018), 25 patients were enrolled and treated from January 2013 to June 2015 at Princess Margaret Cancer Centre in Toronto, Canada (Fig. 1). Patient characteristics are listed in Table 1. A total of 18 patients (72%) were female, with a median age of 54 (range 22–73), with leiomyosarcoma (40%) as the most commonly treated histology. Overall, 8 patients (32%) had one prior line of systemic therapy while 17 (68%) received ENMD-2076 in the first-line setting. At enrollment, 9 (36%) patients had *de novo* metastatic STS, and 16 (64%) had progressive disease in the 6 months prior to enrollment. Post-study systemic therapy was administered in 17 (68%) patients, with an average of 1.52 lines (range 0–4) post progression.

**Table 1:** Patient characteristics (n = 25).

Characteristics		N (%)
Age, median (range)		54 (22–73)
Gender	Female	18 (72%)
	Male	7 (28%)
ECOG PS*	0	13 (52%)
	1	12 (48%)
Histology	Leiomyosarcoma	10 (40%)
	ASPS	3 (12%)
	UPS	3 (12%)
	Angiosarcoma	3 (12%)
	Other^a^	6 (24%)
Number of prior systemic lines of treatment	0	17 (68%)
	1	8 (32%)
Tumor Grade	Low	1 (4%)
	Intermediate	2 (8%)
	High	14 (56%)
	Unknown	8 (32%)

^a^Other = malignant peripheral nerve sheath tumor (1), fibrosarcoma (1), clear cell sarcoma (1), epithelioid sarcoma (1), sarcoma not otherwise specified (1), synovial sarcoma (1).

*Abbreviations –* ASPS, alveolar soft part sarcoma; UPS, undifferentiated pleomorphic sarcoma; ECOG, Eastern Cooperative Oncology Group; PS, Performance status.

### Treatment administration and toxicity

All patients enrolled received study drug and constituted the safety evaluable population. The median number of cycles received was 2 (range 1–10) with a total of 90 cycles administered for the entire cohort. Two patients experienced dose delays (total of 21 doses) for fatigue (one-week delay) and personal reasons (two-week delay). Dose reductions were required in 11 (44%) patients. Treatment was discontinued for disease progression in 21 patients (84%), toxicity in 2 patients (8%), and symptomatic deterioration in 2 patients (8%). Highest grade treatment-related adverse events (trAE) experienced by ≥10% of the safety evaluable population defined as possibly-, probably- or related to study treatment, with those experiencing grade ≥3 toxicities are outlined in Table 2. The most common grade ≥3 toxicity was hypertension (68%) with one patient suffering from subsequent grade 3 posterior reversible leukoencephalopathy that resolved without neurologic sequelae (discontinued from study). Patients with hypertension were managed with antihypertensive medications with no treatment discontinuation indicated. Other common trAE of any grade included fatigue (64%), diarrhea (52%), proteinuria (48%), ALT increase (48%), hypoalbuminemia (48%) dyspepsia (44%), lymphopenia (44%) and nausea (44%). One patient died of colitis during the study which was deemed related to underlying malignancy.

**Table 2:** Highest grade treatment-related Adverse Events (trAE) occurring in ≥10% of patients receiving ENMD-2076.

CTCAE v4.0 classification	Any grade N (%)	Grade 3/4 N (%)
Hypertension	17 (68)	15 (60)
Fatigue	16 (64)	0 (0)
Diarrhea	15 (52)	1 (4)
ALT Increased	12 (48)	2 (8)
Hypoalbuminemia	12 (48)	0 (0)
Proteinuria	12 (48)	0 (0)
Dyspepsia	11 (44)	0 (0)
Lymphopenia	11 (44)	0 (0)
Nausea	11 (44)	0 (0)
Thrombocytopenia	10 (40)	1 (4)
AST Increased	9 (36)	1 (4)
Constipation	8 (32)	0 (0)
Xerostomia	8 (32)	0 (0)
Dizziness	7 (28)	0 (0)
Hyponatremia	7 (28)	1 (4)
Mucositis oral	7 (28)	0 (0)
ALP Increased	6 (24)	0 (0)
Headache	6 (24)	0 (0)
Anemia	5 (20)	1 (4)
White blood cell decreased	5 (20)	1 (4)
Edema	4 (16)	0 (0)
Glucose intolerance	4 (16)	0 (0)
Hematuria	4 (16)	0 (0)
Hypomagnesemia	4 (16)	0 (0)
Vomiting	4 (16)	0 (0)
Abdominal pain	3 (12)	0 (0)
Dysgeusia	3 (12)	0 (0)
Flatulence	3 (12)	0 (0)
Hoarseness	3 (12)	0 (0)
Hyperuricemia	3 (12)	0 (0)
Neutrophil count decreased	3 (12)	1 (4)
Palmar-plantar erythrodysesthesia syndrome	3 (12)	0 (0)
Rash maculo-papular	3 (12)	0 (0)
UTI	3 (12)	0 (0)

^a^Events considered at least possibly related to study treatment. Patients may appear in the table more than once.

*Abbreviations* – CTCAE, Common terminology criteria for adverse events; ALT, alanine aminotransferase; ALP, Alkaline phosphatase; UTI, Urinary Tract Infection.

### Efficacy

**Figure 2:**
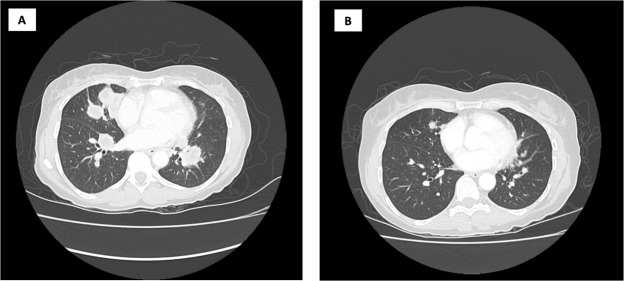
Computed tomography of a patient with undifferentiated pleomorphic sarcoma at cycle 2 (**A**) and cycle 6 (**B**) showing a partial response.

**Figure 3:**
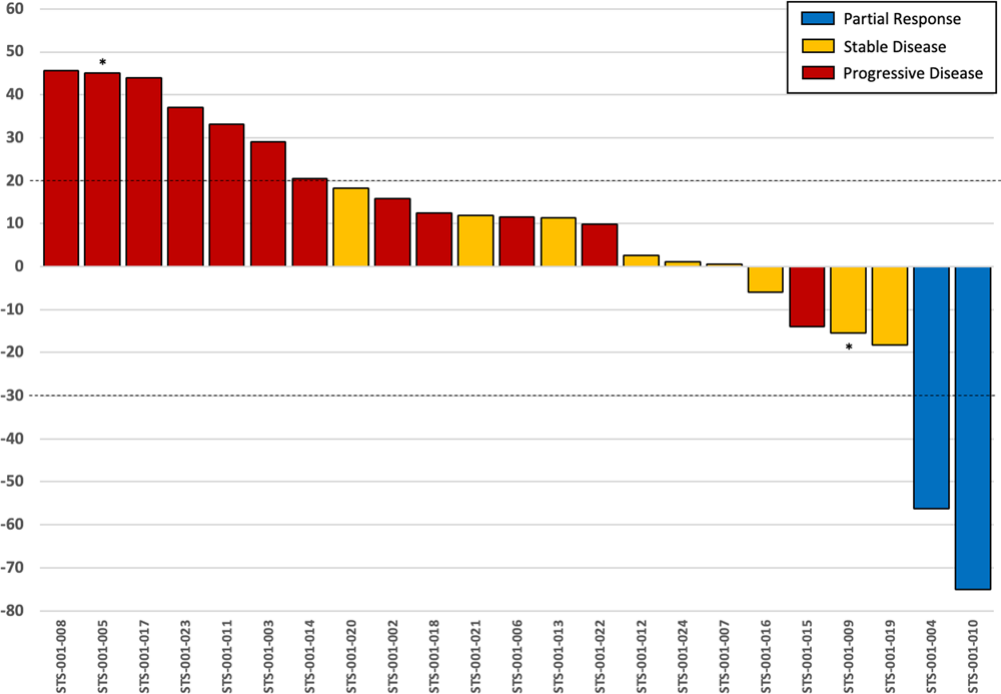
Waterfall plot of patient best response on EMND-2076. * = discontinuation due to toxicity.

**Figure 4:**
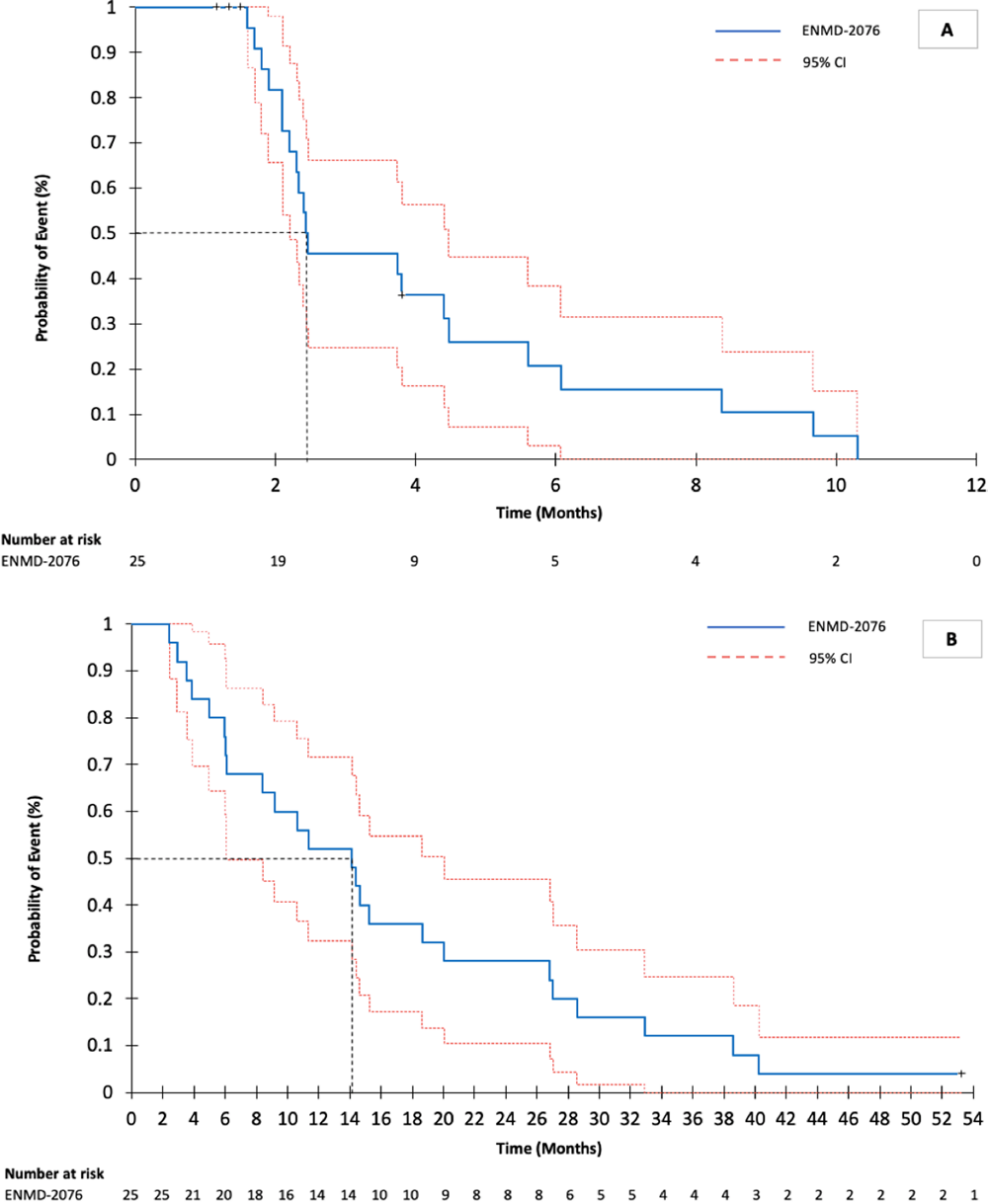
Kaplan-Meier survival curves for patient PFS (A) and OS (B) *Abbreviations*: 95% CI, 95% confidence intervals.

Of 25 patients, 23 (92%) were efficacy evaluable, with a median follow-up of 14 (range 2.2–39.5) months. Final analysis of the phase 2 primary endpoint of 6-month PFS was 20.8% (95% CI: 3.2–38.4), which failed to meet the primary endpoint of ≥40% for further interest. The clinical benefit rate (CBR ≥6-months) was 17% (95% CI: 1.55–33.23) and the ORR was 9% (95% CI: 3.08–20.46) (Table 3). In total, two confirmed partial responses (PR) were observed (9%), one with undifferentiated pleomorphic sarcoma (Fig. 2) and the other with radiation induced angiosarcoma, with response lasting for 10.3 and 6.3 months respectively. Two patients remained on study with stable disease (SD) for a prolonged (>6 months) period of time, one with a malignant peripheral nerve sheath tumor (MPNST; 9.8 months) and one with uterine leiomyosarcoma (8.5 months). A waterfall plot of patient best response is shown in Fig. 3. Median PFS was 2.5 months (95% CI 2.20–2.47), and median OS was 14.1 months (95% CI 6.07–20.07). Kaplan-Meier curves for PFS (Fig. 4A) and OS (Fig. 4B) are shown for the full study cohort.

**Table 3:** Clinical outcomes of patients receiving ENMD-2076.

**Median number of cycles (range)**		2 (1–10)
**≥6-month PFS (%)**		20.8% (95% CI: 3.2–38.4)
Best Response^a^; N (%)	PR^b^	2 (9%)
	SD^c^	8 (35%)
	PD	13 (56%)
ORR (%)	CR + PR	9% (95% CI: 1.55–33.23)
CBR (%)	CR + PR + SD ≥6 months	17% (95% CI: 3.08–20.46)
Median OS		14.1 months (95% CI 6.07–20.07)
Median PFS		2.5 months (95% CI 2.20–4.47)

^a^23 of 25 patients were evaluable for response.

^b^Tissue types with PR were, angiosarcoma and undifferentiated pleomorphic sarcoma.

^c^Tissue types with SD ≥6-months were, leiomyosarcoma and malignant peripheral nerve sheath tumor (MPNST).

*Abbreviations* – PFS, progression free survival; PR, partial response; SD, stable disease; PD, progression of disease; ORR, Overall Response Rate; CBR, Clinical Benefit rate; OS, overall survival.

### Molecular Associative Studies

Tumor samples from 4 patients benefiting from study drug (1 PR; 2 prolonged SD >6 months; 1 SD <6 months, discontinuing from study due to toxicity) and 8 not benefiting (PD on first restaging evaluation) were subjected to DNA extraction and whole exome sequencing (WES) of 4813 genes. One patient who experienced a confirmed PR (56.2% reduction) did not have adequate tissue for sequencing and was not included in the analysis.

Overall, 8129 unique exonic non-synonymous variants were identified. Tumors from patients benefiting from therapy had a numerically lower number of genetic variants compared to those not benefiting (mean 7704 vs 7948, p = 0.12). Genes that were found to be altered in a majority of benefiting (≥3 out of 4; ≥75%) and not benefiting (≥6 out of 8; ≥75%) patients are presented in Table 4. Only two unique variants were identified in the *TP53* gene: two missense variants (p.P33A and p.P72A) in 7/8 (87.5%) not benefiting patients; and 4/4 (100%) benefiting patients. Another variant (G649T) was a nonsense mutation seen only in one not benefiting patient (data not shown). In patients not benefiting from ENMD-2076, mutually exclusive mutations were identified in 7 genes related to scaffolding proteins (*RSPH4A;* 8/8, p = 0.002), phosphatidylinositol signaling (*PLC-E1*, 7/8; p = 0.01), WNT pathway (*LINS*; 6/8; p = 0.03), hedgehog signaling (*EVC*; 6/8; p = 0.03), G-protein coupled adhesion receptors (*GPR98*; 6/8; p = 0.03), and extracellular matrix remodeling (*ADAMTS18;* 6/8; p = 0.03). Mutation in *PLC-E1* resulted in a frameshift mutation. Of patients deemed benefiting (n = 4) from ENMD-2076, mutually exclusive mutations were identified in 7 genes related to carbohydrate metabolism (*PDHX;* 4/4; p = 0.002), kinetochore scaffolding (CASC5/*KNL1;* 3/4; p = 0.018), cholesterol homeostasis (*SREBF2*; 3/4; p = 0.018; *LPL*; 3/4; p = 0.018), angiogenesis (*PTPRB;* 3/4; p = 0.018), and apoptosis (*TP53-I3*; 3/4; p = 0.018).

**Table 4:** Mutually exclusive genetic alterations occurring in ≥75% of the not benefiting (top) and benefiting (bottom) cohorts.

Gene	Variation	Mutation	Number Not Benefiting (n = 8)	Fisher’s Exact; p-value	Cellular Pathway Relationship
**Mutations in not benefiting patients**					
RSPH4A	SNV	exon4:c.T1766C:p.L589P	8	0.002	Ciliary scaffold protein
PLC-E1	SNV	exon19:c.G3800C:p.R1267P exon20:c.G4676C:p.R1559P exon20:c.G4724C:p.R1575P	7	0.01	Calcium signaling pathway; Inositol phosphate metabolism
EVC	SNV	exon12:c.G1727A:p.R576Q	6	0.03	Hedgehog Signaling. Ellis Van Creveld syndrome
LINS	SNV	exon5:c.G1216A:p.V406M	6	0.03	WNT signaling pathway regulator
ADAMTS18	SNV	exon22:c.G3476C:p.S1159T	6	0.03	Disintegrin metalloproteinase
GPR98	SNV	exon82:c.G17626A:p.V5876I	6	0.03	Adhesion receptors; G-protein coupled receptor
**Mutations in benefiting patients**					
**Gene**	**Variation**	**Mutation**	**Number Benefiting (n = 4)**	**Fisher’s Exact; p-value**	**Cellular Pathway Relationship**
PDHX	SNV	c.A301G	4	0.002	Metabolism of carbohydrates. Pyruvate Dehydrogenase
KNL1	SNV	exon10:c.A4339G:p.T1447A exon11:c.A4417G:p.T1473A	3	0.018	Cell Cycle, Mitotic. Kinetochore Scaffold
SREBF2	SNV	exon14:c.G2580C:p.R860S	3	0.018	Cholesterol homeostasis
LPL	SNV	exon9:c.C1421G:p.S474X	3	0.018	Metabolism of lipids and lipoproteins
PTPRB	SNV	exon6:c.T1245G:p.D415E	3	0.018	Adherens junction; Angiogenesis
		exon8:c.T1899G:p.D633E			
TP53-I3	SNV	exon4:c.A688G:p.T230A	3	0.018	p53 signaling pathway; apoptosis

*Abbreviations* – SNV, single nucleotide variant; NA, not available.

## Discussion

This study demonstrated low level activity of ENMD-2076 in advanced STS. Although the 6-month PFS (20.8%) in the efficacy evaluable population refuted the null hypothesis (≤15%), the study did not meet its primary endpoint of ≥40% to demonstrate activity of interest. The short median PFS (2.5 months) and OS (14.1 months) are consistent with existing STS literature for ineffective therapy/placebo^4,14^. This discrepancy may be explained by a non-representative cohort of STS. For example, in this study 72% of patients were female, no patients with liposarcoma were enrolled (known activity to AURKA inhibitors)^15^, and almost half of the patients had leiomyosarcoma histology (compared to 30% in the PALETTE^4^ study) which may have influenced outcomes. However, in keeping with other TKI studies^4,16^ a considerable number of tumors expressed high grade histology (56%). In general, soft tissue sarcomas represent a heterogeneous group with more than 70 histologic subtypes. This study once again raises a key challenge with STS clinical trials, as it is increasingly recognized that different subtypes exhibit variability in their sensitivity to systemic treatments^17–19^.

Interestingly, activity was identified in two patients with objective partial response and another two with prolonged stable disease. One of the documented partial responses occurred in a patient with radiation induced angiosarcoma. A study with another AURKA inhibitor, alisertib in advanced STS recently reported an ORR of 2.8%, with two partial responses occurring in patients with angiosarcoma^20^. ENMD-2076 has both anti-angiogenic activity through inhibition of VEGFR pathway, as well as mitotic spindle inhibition via the AURKA pathway. While anti-angiogenic agents demonstrate modest response rates in patients with angiosarcoma^3,21,22^, paclitaxel, a microtubule depolymerization inhibitor has demonstrated considerable activity in this STS subtype^23^. Prolonged stable disease was also demonstrated in one patient with MPNST (9.5 months). Similar results of prolonged stable disease have been shown by Dickson, *et al*.^20^ with twelve-week PFS rates of 60% for MPNST. Activity for EMND-2076 in this subtype is biologically plausible, as pre-clinical evaluation of neurofibromatosis type 1 (NF1) tumors demonstrated MPNST subtypes as over expressing VEGF and having higher vessel density relative to other NF-1 tumor types^24^. Other trials evaluating bevacizumab (anti-VEGF) in combination with everolimus (mTOR inhibitor) have demonstrated only modest clinical benefit (CBR = 12%)^25^, although sunitinib (VEGFR-TKI) is also currently being investigated in MPNST (NCT 01402817).

ENMD-2076 was generally well tolerated in trial participants and the safety profile is consistent with other multi-kinase inhibitors and prior safety data for this agent^10,13^. Hypertension was the most common grade ≥3 adverse event, with rare cases of other grade ≥3 toxicity. The most common toxicities, diarrhea and fatigue, occurred in 52% and 64% of patients and was mostly grade 1–2. Treatment was discontinued for adverse events in only 2 patients, reinforcing its general tolerability.

It has been reported that AURKA phosphorylates p53 resulting in its poly-ubiquitination by MDM2 and inhibition of apoptotic activity^26^. Thus, AURKA inhibition can promote accumulation of p53 and cell cycle arrest^27^. *TP53* mutation and p53 over-expression were found to be associated with sensitivity to ENMD-2076 in triple-negative breast cancer cell-lines^13^. Our exploratory analyses, using whole exome DNA sequencing (WES) in four patients benefiting and eight not benefiting patients, did not support the role of *TP53* mutations as a predictive biomarker, possibly due to small sample size and high prevalence of variants in this gene. Noteworthy is the presence of a point mutation in the coding region of the p53 inducible protein (*TP53-I3*), occurring in three out of four benefiting patients.

Another intriguing finding is the presence of *PTPR*B (protein tyrosine phosphatase receptor type B) missense mutation (T1245G) in three out of four benefiting patients. PTPRB serves as a negative regulator of Tie2, a receptor implicated in the angiogenic pathway^28^. *In-vitro* models have shown that PTPRB inhibition increased angiogenesis while pharmacologic VEGF inhibition in PTPRB-silenced cell lines reduced angiogenesis^29^. *PTPRB* mutations are considered rare in solid malignancies, nevertheless they were present in 26% of 39 angiosarcoma tumors sequenced. This clinical information supports further investigation of PTPRB as a potential biomarker of response to anti-VEGF therapy.

The KMN network protein, KNL-1 involved in the protein architecture of kinetochores^30^, was also identified by WES as having point mutations in 3 out of 4 benefiting patients. KNL-1 undergoes phosphorylation by Aurora B kinase (AURKB), however the function of *the A4339G/A4417* mutations on *KNL-1* activity are unknown. ENMD-2076 is also 24-fold more AURKA selective relative to AURKB^9^, possibly limiting the implication of *KNL-1* mutations.

One limitation of this study is that only a select number of tissue specimens were evaluated by WES. Due to low response rates, further sample evaluation would likely not yield additional insights but may limit conclusions. Three patients deemed benefiting from ENMD-2076 in molecular analyses were synonymous with the CBR definition (≥6-months) of benefit. However, 1 patient with an SD who was benefiting clinically from ENMD-2076, came off study early (<6 months) due to a serious adverse event (reversible posterior leukoencephalopathy syndrome). WES of this patient was of particular interest due to their toxicity profile.

Overall, ENMD-2076 is well tolerated in patients with advanced STS. Although this study did not meet its primary endpoint, this agent may be active in specific STS subtypes such as angiosarcoma, and the ORR is comparable to results with other multi-targeted tyrosine kinase inhibitors in STS. The role of *PTPRB* and *p53* pathway alterations in sarcomas receiving multi-targeted TKIs should be explored further.

## Patients and Methods

### Study design

This single center, single arm, phase II study evaluated ENMD-2076 continuous oral monotherapy given at a daily dose of 275 mg/day on a 28-day cycle until disease progression or unacceptable toxicity in patients with metastatic STS. The primary endpoint was 6-month PFS rate defined as the percentage of patients remaining on trial at the 6-month timepoint. PFS was defined as the time from first day of study treatment to first disease progression or death due to any cause. Secondary endpoints included safety and tolerability, defined by the frequency and severity of adverse events using the Common Terminology Criteria for Adverse Events (CTCAE) version 4.0. Clinical benefit rate (CBR = complete response [CR] + partial response [PR] + stable disease [SD] ≥ 6-months) and objective response rate (ORR = CR + PR) were evaluated using RECIST v1.1. Overall survival (OS) was defined as the time from first day of study treatment to the date of death from any cause.

Radiographic and clinical tumor assessments occurred at baseline and every two cycles. Patients underwent review of symptoms, physical exam, vital signs evaluation, Eastern Cooperative Oncology Group (ECOG) performance status assessment, laboratory investigations (complete blood count, chemistry, coagulation test, urinalysis, pregnancy test [if appropriate]) and electrocardiogram (ECG) prior to study entry and at fixed intervals while on study (S1: Appendix Table 1). MUGA scan or ECG as well as collection of archival tumor specimens were performed at baseline.

### Eligibility criteria

Patients were eligible for study entry if they had histologically confirmed metastatic STS (with the exception of gastrointestinal stromal tumor) with no more than one line of prior systemic therapy and at least one unidimensional measurable lesion according to RECIST v1.1 criteria. Other inclusion criteria included age ≥18, ECOG performance status of 0 or 1, enrollment at least three weeks from any surgery or anticancer therapy (irradiation, chemotherapy or biological agents), left ventricular ejection fraction (according to MUGA or ECG) equal to or greater than the institution lower limit of normal within a month prior to start of study, adequate hematopoietic (absolute neutrophil count ≥1,500 cells/mm^3^, platelets ≥100,000/mm^3^, hemoglobin ≥9.0 g/dL), hepatic (AST and ALT ≤2.5 times upper limit of normal [ULN] or <5 X ULN if liver metastases are present, total bilirubin ≤1.5 x ULN), and renal (serum creatinine <1.5 X ULN or calculated creatinine clearance ≥50 mL/min, ≥2+ proteinuria) function. Enrollment exclusions included women who were pregnant or nursing, specific co-existing uncontrolled medical condition (including active infections or bleeding), uncontrolled hypertension (systolic blood pressure >150 mmHg or diastolic blood pressure >100 mmHg), active cardiac disease in the previous 6 months, psychiatric illness, progressive/untreated brain metastases, other active malignancy within 5 years, or gastrointestinal abnormalities that would impair the administration or absorption of oral drugs.

The protocol was approved by the Princess Margaret Cancer Centre institutional review board(s) and conforms to the Helsinki Declaration. All participants provided written informed consent prior to performing study related procedures or obtaining archival tissues (ClinicalTrials.gov registration: NCT01719744; 01/11/2012).

### Molecular associative analyses

An exploratory analysis of archival tissue for tumor genomic alterations was conducted in an attempt to identify potential biomarkers of benefit or resistance to ENMD-2076. Formalin-fixed paraffin embedded (FFPE) archival tumor samples from patients stratified as benefiting from study drug (defined as PR or SD) vs not benefiting (PD or SD lasting less than 3 months) were subjected to DNA extraction and deep sequencing of >4800 genes (Illumina Miseq). Variants were annotated with previously known drug response data (ClinVar and PharmGKB)^31^, pathway analyses (performed using DAVID Functional Annotation Bioinformatics; https://david.ncifcrf.gov/), and variant type (nonsense, frameshift, splice site, and missense changes within coding regions).

### Statistical analysis

Sample size for this trial was calculated for single agent ENMD-2076 using a null hypothesis of 6-month PFS of ≤15% indicating lack of compound interest, and an alternative hypothesis of 6-months PFS of ≥40% indicating further interest. Based on a significance level of 10% and a power of 90%, a sample size of ≥21 patients was determined. Safety analyses were performed in the population of patients that received at least 1 dose of study treatment. Efficacy analyses were done in all patients who had at least one post-baseline radiographic assessment of target, non-target, or new lesions. Secondary endpoints of CBR ≥6-months and ORR as assessed by RECIST v1.1 were represented descriptively with 95% confidence intervals (95%CI). PFS and OS were evaluated using the Kaplan-Meier method. Association of potential genomic biomarkers with response profiles was performed using 1-sided Fisher’s Exact testing. Statistical analyses were performed using XLSTAT® v2018.1 and SPSS® software v24.0.

## Supplementary information

Appendix Table 1

## Data Availability

The datasets generated duration and/or analyzed during the current study are not publicly available due to patient privacy laws (Personal Information Protection and Electronic Documents Act [PIPEDA]) but are available from the corresponding author on reasonable request.
