# Supplementary material for: A phase II study of ENMD-2076 in advanced soft tissue sarcoma (STS)
Source: Sci Rep. 2019 May 14;9:7390. doi: 10.1038/s41598-019-43222-6 (PMC6517396; doi:10.1038/s41598-019-43222-6)
Supplement: Supplementary file 1 — Appendix Table 1 [file 41598_2019_43222_MOESM1_ESM.docx]

**A phase II study of ENMD-2076 in advanced soft tissue sarcoma (STS)**

**Authors:** Zachary Veitch^1,2^, Alona Zer^1,2^, Herbert Loong^1,2^, Samer Salah^1,2^, Maryam Masood^1^, Abha Gupta^1,2^, Penelope A. Bradbury^1,2^, David Hogg^1,2^, Andrew Wong^3^, Rita Kandel^3,4,5^, George S. Charames^3,4,5^, Albiruni R Abdul Razak^1,2^,

**Affiliation:**

(1) Princess Margaret Cancer Centre, Toronto, Canada

(2) Department of Medicine, University of Toronto, Toronto, Canada

(3) Department of Pathology and Lab Medicine, Mount Sinai Hospital, Toronto, Canada

(4) Department of Lab Medicine and Pathobiology, University of Toronto, Toronto, Canada

(5) Lunenfeld-Tanenbaum Research Institute, Sinai Health System, Toronto, Canada

**S1: Appendix Table 1** – schedule of evaluations for patients receiving ENMD-2076

| **Measurement/Treatment** | **Screening** | **Treatment Day (Cycle 1)** | | | | **Treatment Day (subsequent cycles)** | | | |
| --- | --- | --- | --- | --- | --- | --- | --- | --- | --- |
|  | **Days**  **–28 to –1** | **Day 1** | **Day 8** | **Day 15** | **Day 22** | **Day 1 of each subsequent cycle (Day 29)** | **Day 15 (> Cycle 6; does not have to be done for subsequent Cycles)** | **End of Cycle 6/180 days** | **End of Study Drug Administration** |
|  |  |  |  |  |  |  |  |  |  |
| Inclusion/Exclusion | X |  |  |  |  |  |  |  |  |
| Informed Consent | X |  |  |  |  |  |  |  |  |
| Medical History | X |  |  |  |  |  |  |  |  |
| MUGA/ ECHO (repeat as indicated) | X |  |  |  |  |  |  |  |  |
| Physical Examination | X |  |  |  |  | X |  |  | X |
| Vital Signs (HR, temp, RR, BP) | X | X | X | X | X | X | X |  | X |
| ECOG Performance Status | X | X |  |  |  |  |  |  |  |
| Hematology | X | X | X | X | X | X | X |  | X |
| Clinical Chemistry | X | X | X | X | X | X | X |  | X |
| Coagulation Tests | X | X |  |  |  | X |  |  |  |
| Urinalysis | X | X | X | X | X | X | X |  | X |
| Pregnancy Test | X | X |  |  |  | X |  |  | X |
| ENMD-2076 treatment once daily |  | --X-- | --X-- | --X-- | --X-- | --X-- | --X-- |  |  |
| Electrocardiogram (locally read) | X | X |  |  |  | X |  |  |  |
| Radiographic Tumor Evaluation | X |  |  |  |  | X (even cycles) |  | X | X |
| Tissue block/slides | X |  |  |  |  |  |  |  |  |
| Optional Fresh Tumour Biopsy | X | X |  | X |  |  |  |  |  |
| AE Monitoring |  | --X-- | --X-- | --X-- | --X-- | --X-- | --X-- |  | --X-- |
| Concomitant Medications | X | --X-- | --X-- | --X-- | --X-- | --X-- | --X-- |  | --X-- |

***Abbreviations*** – ECOG, Eastern Cooperative Oncology Group; RR, Respiratory rate; HR, Heart Rate; BP, Blood pressure; AE, Adverse Event.
